# Hommage au Professeur Michel Dumas (1934-2025)

**DOI:** 10.48327/mtsi.v6i2.2026.846

**Published:** 2026-04-21

**Authors:** Amadou Gallo DIOP, Pierre-Marie PREUX

**Affiliations:** 1 Ancien chef du Département de neurosciences, Université de Dakar; Ancien administrateur de la Fédération mondiale de neurologie; Membre de l’Académie nationale des sciences, Sénégal; 2 Directeur de l’Institut d’épidémiologie et de santé mondiale - Michel Dumas, Limoges; Directeur de l’EpiMaCT, Inserm U1094, IRD UMR270, Limoges, France

**Figure 1:**
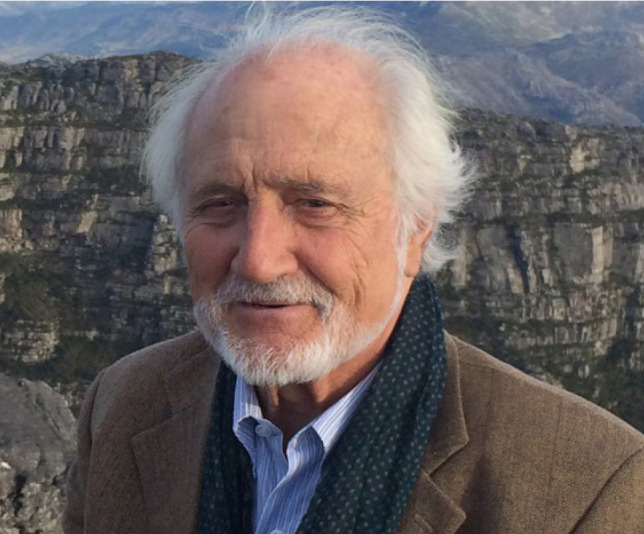
Professeur Michel Dumas, Le Cap, lors du Deuxième Congrès sur l’épilepsie en Afrique, 2014 (crédit photo : P.-M. Preux)

Michel Dumas nous a quittés le 8 novembre 2025. Neurologue, neuroépidémiologiste, tropicaliste, il était membre correspondant de l’Académie nationale de médecine, professeur émérite de l’Université de Limoges, et a été chef du service de neurologie du CHU. Il est le fondateur de l’Institut d’épidémiologie et de neurologie tropicale, dont il a assuré la direction pendant plus de 25 ans. Il laisse une œuvre immense dans le domaine de la médecine tropicale.

Né en 1934 en Afrique de l’Ouest, il a grandi dans une double culture, humaniste et universaliste. Élève du Professeur Henri Collomb à Dakar, il a été chef du service de neurologie du CHU de Dakar et a fortement contribué à structurer la neurologie clinique et universitaire de l’Afrique de l’Ouest.

De retour en France à Limoges en 1976, il fonde l’Institut d’épidémiologie neurologique et de neurologie tropicale (IENT). Ce fut l’un des premiers instituts universitaires français explicitement tourné vers la médecine tropicale neurologique, avec une triple vocation : la recherche, la formation, et la coopération internationale. Visionnaire, il y intègre très tôt les dimensions populationnelles et épidémiologiques qui deviendront centrales dans le développement ultérieur de l’Institut et de son unité de recherche.

Le Professeur Dumas s’est intéressé très tôt à l’épidémiologie des maladies neurologiques dans les pays tropicaux. Il a contribué à définir les bases de la neurologie tropicale moderne, notamment par ses travaux de terrain conduits en Afrique, en Asie du Sud-Est et en Amérique latine. Il est l’auteur de plus de 500 publications et chapitres de livres.

Largement reconnu à l’international, il a reçu en 2015 la médaille d’honneur pour services rendus à la neurologie mondiale, décernée par la Fédération mondiale de neurologie. Il a été distingué par plusieurs pays africains et asiatiques pour son engagement dans la formation médicale spécialisée.

Mais son impact a sans doute été encore plus important sur le plan humain et institutionnel. Il a accompagné la formation de plusieurs centaines de neurologues et de professionnels de santé du Sud. Il a fait de Limoges un centre majeur de formation et de recherche en neurologie tropicale francophone.

C’était un homme de convictions, d’action, et de transmission. Il a porté très tôt la vision d’une médecine équitable, rigoureuse et fraternelle. À travers l’Institut qu’il a fondé, il a également aidé d’autres disciplines à s’implanter et à coopérer : hématologie, médecine interne, gériatrie, psychiatrie, etc.

Sa contribution à la médecine tropicale ne réside pas seulement dans ses travaux scientifiques ou dans les formations dispensées. Elle réside surtout dans sa capacité à créer des institutions durables, à faire émerger de nouveaux leaders scientifiques du Sud, et à inscrire la neurologie tropicale dans le champ plus vaste de la santé mondiale. Il avait accepté, non sans réticence et avec beaucoup d’humilité, que son nom soit donné à l’Institut qu’il avait fondé, rebaptisé en septembre 2025 « Institut d’épidémiologie et de santé mondiale - Michel Dumas ». Il répétait d’ailleurs que l’on devait se présenter sans titres : nous sommes d’abord des hommes avant d’être médecin ou professeur.

**Figure 2:**
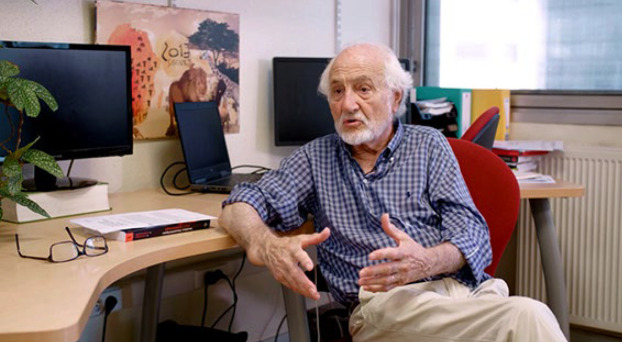
Professeur Michel Dumas, Limoges, lors d’un tournage d’une vidéo d’éducation sur l’épilepsie, 2020 (crédit photo : Émilie Auditeau)

Michel Dumas est resté fidèle à l’Institut jusqu’à ses tout derniers jours : on le voyait encore arriver le matin, saluer chacun avec chaleur, puis s’installer à son bureau, musique classique en fond sonore, pour trier ses archives et préparer la transmission. Sa porte était toujours ouverte, et il trouvait le temps de déposer sur un bureau un dossier utile, parfois accompagné de « *l’Éloge de la fatigue* » de Robert Lamoureux, qu’il aimait tant et qui disait si bien sa conception du travail comme responsabilité envers les autres. Il continuait à se déplacer, à accueillir lui-même étudiants et collègues venus d’Afrique ou d’ailleurs, à la gare de Limoges comme autrefois, et à suivre le destin de chacun avec une attention fidèle. Dans son jardin, il aimait partager des moments simples avec les enfants de ses collègues. Cette présence discrète mais constante, faite d’exigence, de bienveillance et d’engagement, résume peut-être mieux que tout discours la manière dont il concevait la médecine, la recherche et la transmission. Il avait le don rare de renforcer la confiance de ses élèves tout en leur transmettant l’humilité du savoir.

Son héritage se perpétue dans les nombreuses vies qu’il a marquées, les institutions qu’il a construites, et les idées qu’il a défendues. Merci pour tout, Cher Maître, au nom de plusieurs générations d’élèves de vos élèves.

**Figure 3:**
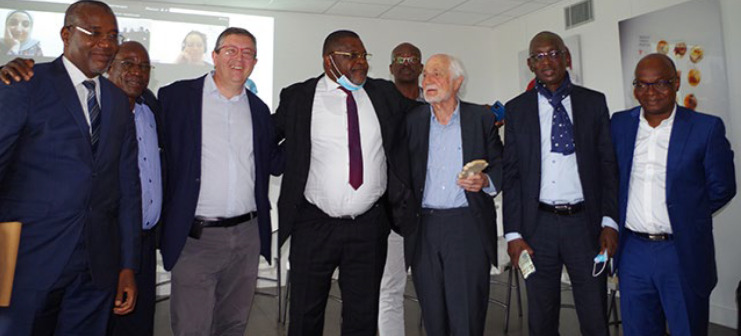
Professeur Michel Dumas et quelques-uns de ses élèves, Limoges, à l’occasion des 40 ans de l’Institut d’Épidémiologie et de Neurologie Tropicale, 2022. De gauche à droite : Mouhamadou Diagana (Mauritanie), Pascal Mbelesso (République Centrafricaine), Pierre-Marie Preux (France), Edgard Ngoungou (Gabon), Athanase Millogo (Burkina Faso), Michel Dumas (France), Amadou Gallo Diop (Sénégal), Dismand Houinato (Bénin) (crédit photo : Émilie Auditeau)
