# Supplementary material for: Tribute to Professor Michel Dumas (1934–2025)
Source: Med Trop Sante Int. 2026 Apr 21;6(2):mtsi.v6i2.2026.846. [Article in French] doi: 10.48327/mtsi.v6i2.2026.846 (PMC13423295; doi:10.48327/mtsi.v6i2.2026.846)
Supplement: Related texts

# Research article: Tribute to Professor Michel Dumas (1934–2025)

**Figure 1:** Professor Michel Dumas, Cape Town, at the Second African Epilepsy Congress, 2014 (photo credit: P.-M. Preux)

Michel Dumas passed away on November 8, 2025. He was a neurologist, neuroepidemiologist, and tropical medicine specialist. He was a corresponding member of the National Academy of Medicine and a professor emeritus at the University of Limoges. He also served as head of the neurology department at the University Hospital. He founded the Institute of Epidemiology and Tropical Neurology and led it for over 25 years. He leaves behind an immense body of work in tropical medicine.

Born in West Africa in 1934, he grew up immersed in both a humanistic and a universalist culture. He studied under Professor Henri Collomb in Dakar and served as head of the neurology department at the University Hospital of Dakar, making significant contributions to the development of clinical and academic neurology in West Africa.

Upon returning to Limoges, France, in 1976, he founded the Institute of Neurological Epidemiology and Tropical Neurology (IENT). It was one of the first French university institutes explicitly focused on tropical neurological medicine, with a threefold mission of research, training, and international cooperation. A visionary, Professor Dumas incorporated population-based and epidemiological dimensions into the Institute at a very early stage. These dimensions would become central to the subsequent development of the institute and its research unit.

Professor Dumas took an early interest in the epidemiology of neurological diseases in tropical countries. He helped lay the foundations of modern tropical neurology, notably through his fieldwork in Africa, Southeast Asia, and Latin America. He is the author of more than 500 publications and book chapters.

He is widely recognized internationally and received the Medal of Honor for his services to global neurology in 2015 from the World Federation of Neurology. Several African and Asian countries have honored him for his commitment to specialized medical training.

However, his impact was undoubtedly even greater on a human and institutional level. He mentored several hundred neurologists and healthcare professionals from the Global South. He established Limoges as a leading center for training and research in Francophone tropical neurology.

He was a man of conviction, action, and mentorship. From early on, he championed the vision of equitable, rigorous, and compassionate medicine. Through the institute he founded, he helped other disciplines take root and collaborate, including hematology, internal medicine, geriatrics, and psychiatry.

His contributions to tropical medicine extend beyond his scientific work and the training he provided. Above all, it lies in his ability to create sustainable institutions, foster new scientific leaders from the Global South, and place tropical neurology within the broader field of global health. With great humility and some reluctance, he agreed to have his name given to the institute he founded, which was renamed the “Michel Dumas Institute of Epidemiology and Global Health” in September 2025. Moreover, he often said that one should introduce oneself without titles because we are human beings first and foremost, before being doctors or professors.

**Figure 2:** Professor Michel Dumas, Limoges, during the filming of an educational video on epilepsy, 2020 (photo credit: Émilie Auditeau)

Michel Dumas remained loyal to the Institute until his last days. One could still see him arrive in the morning and greet everyone warmly. Then he would settle into his office with classical music playing in the background to sort through his archives and prepare for the handover. His door was always open, and he always found time to place a useful file on someone’s desk. Sometimes he would accompany it with Robert Lamoureux’s “In Praise of Fatigue,” which he loved so much and which so aptly expressed his view of work as a responsibility toward others. He continued to travel and personally welcome students and colleagues from Africa and elsewhere at the Limoges train station, as he had in the past. He followed each person’s journey with faithful attention. In his garden, he enjoyed spending time with his colleagues’ children. His discreet yet constant presence, marked by high standards, kindness, and dedication, perhaps sums up the way he viewed medicine, research, and the transmission of knowledge better than any speech could. He had the rare gift of bolstering his students’ confidence while instilling in them the humility of learning.

His legacy lives on in the many lives he touched, the institutions he built, and the ideas he championed. Thank you for everything, dear mentor, on behalf of several generations of your students and their own students.

**Figure 3:** Professor Michel Dumas and some of his students, Limoges, on the occasion of the 40th anniversary of the Institute of Epidemiology and Tropical Neurology, 2022. From left to right: Mouhamadou Diagana (Mauritania), Pascal Mbelesso (Central African Republic), Pierre-Marie Preux (France), Edgard Ngoungou (Gabon), Athanase Millogo (Burkina Faso), Michel Dumas (France), Amadou Gallo Diop (Senegal), Dismand Houinato (Benin) (photo credit: Émilie Auditeau)
